# Making the right software choice for clinically used equipment in radiation oncology

**DOI:** 10.1186/1748-717X-9-145

**Published:** 2014-06-23

**Authors:** Hilke Vorwerk, Klemens Zink, Daniela Michaela Wagner, Rita Engenhart-Cabillic

**Affiliations:** 1 Radiotherapy and Radiation Oncology, University Hospital Marburg, Baldingerstrasse, Marburg 35043, Germany; 2 Technische Hochschule Mittelhessen, Wiesenstraße 14, Giessen 35390, Germany; 3 Radiotherapy and Radiation Oncology, University Hospital Göttingen, Robert-Koch-Strasse 40, Göttingen 37099, Germany

**Keywords:** Treatment planning system, ROKIS, High precision radiotherapy, Software choice, Technical applicability, Radiation therapy

## Abstract

The customer of a new system for clinical use in radiation oncology must consider many options in order to find the optimal combination of software tools. Many commercial systems are available and each system has a large number of technical features. However an appraisal of the technical capabilities, especially the options for clinical implementations, is hardly assessable at first view.

The intention of this article was to generate an assessment of the necessary functionalities for high precision radiotherapy and their integration in ROKIS (Radiation oncology clinic information system) for future customers, especially with regard to clinical applicability. Therefore we analysed the clinically required software functionalities and divided them into three categories: minimal, enhanced and optimal requirements for high conformal radiation treatment.

## Introduction

Technical functionality of all soft- and hardware components is a prerequisite for the application of high conformal radiation treatment. Most considerations include technical functionalities but not the clinical practicability. Hence the available tools are mostly limited in their applicability and sometimes, because of the absence of implementation, not applicable at all. The most important consideration is whether it is possible to implement the technical functionalities or not. Therefore we summarized the available technical functionalities and analysed them according to their clinical practicability. We focused on the functionality of the treatment planning system, the patient verification at the accelerator and the ROKIS system because these factors predominantly contribute to the clinical applicability of high precision radiotherapy. This study should assist the reader in making the right software and hardware choice for the implementation of highly conformal radiotherapy in the daily clinical practice.

## Methods and materials

Basic functionality is a prerequisite for the application of highly conformal radiation treatment. Additional tools provide increased requirements, for example safer or faster treatment [1]. In order to identify the features requested, we generated a list based on all features available from a larger radiation oncology vendors. Then, we classified all functionalities into three groups:

(1) minimal requirements for high conformal radiation treatment

(2) enhanced requirements for high conformal radiation treatment (for example faster and/or safer treatment)

(3) optimal requirements for high conformal radiation treatment (best possible features)

The tools, which establish the basic requirements for high conformal radiotherapy for nearly all patients of a clinical department have been analyzed in this article. In our considerations, we included high precision therapy for different clinical cases such as head or neck region as well as high dosage treatment of the prostate. We did not include considerations for special areas such as cranial or extra-cranial radiosurgery or brachytherapy.

## Results

The analysis of the features in the above mentioned groups are summarised in Table 1, Table 2, Table 3, Table 4, and Table 5. For clarity the data were divided into three classifications safety, accuracy and efficiency (Table 1, Table 2 and Table 3). Thereby, the data import, registration and structure contouring influence all three categories. The dicom coordinates from the CT must either be transformed before being imported into the planning system or a new origin must be defined in the treatment planning system, which can result in errors. Therefore the optimal solution is the automatic transformation, either before or directly after the import into the treatment planning system. The CT software and the infrastructure often limit this possibility and the treatment planning systems are repeatedly not able to compensate for this problem. Most of the different contouring tools are basic tools; correction tools and 3D options are enhanced and optimal requirements. Most notably, transferring and copying structures between different data sets is very important for high precision radiotherapy. The demand on the planning system is extensive concerning accuracy as well as safety aspects. (Table 1, Table 2 and Table 3). Special focus should be set on the basic minimal requirement, which is the ability to create a sum of different data sets. Most ROKIS systems offer the requested tools, but the integration of the various existing tools into one system is still under development. Patient verification is the system with the highest demand on new tools in the future, especially for the clinical implementation of single features. Accuracy and safety are most important for the clinical applicability of this feature.

**Table 1:** Requirements for safety

**Data import, data registration and structure contouring**		
**Minimal**	**Enhanced**	**Optimal**
Import of all CT data with dicom coordinates	Import of all CT data with transposition of the dicom coordinates (lower error source)	Structures can be digitally locked by selected users
Import and export of treatment plans in dicom format	Registration based on dicom coordinates	
	Registration is digital lockable	
	Structures, which are connected to a treatment plan, can’t be changed (only copies or new structures can be created)	
**Requirements for treatment planning**		
**Minimal**	**Enhanced**	**Optimal**
Collapse cone or equivalent algorithm for the dose calculation of photons	Monte Carlo or equivalent algorithm for the dose calculation of photons	IMRT optimization with Monte Carlo or equivalent algorithm
Monte Carlo based radiation head model	IMRT optimization with a Collapse cone or equivalent algorithm	Conventional IMRT optimization comprises collimator and gantry rotation
Create sum plans of treatment plans calculated on rigid registered data sets	IMRT optimization with a direct aperture calculation	Volumetric IMRT optimization comprises collimator rotation and segment selection
Comparison of different treatment plans (calculated on any data sets) with simultaneous display of the isodose distributions, DVH and BEV	Monte Carlo or equivalent algorithm for the dose calculation of electrons	Creating of optimal DRR (DRR templates producible and manual adjustable)
Creation of check sum every working day (control of beam data)	More than one reference dose and point can be applied to a treatment plan (e.g. integrated boost)	
**Requirements for ROKIS**		
**Minimal**	**Enhanced**	**Optimal**
Import and export of all data in general readable dicom format (structures, treatment plans, CT/MRI/PET/… data sets, isodoses, verification images such as planar view images or CBCT data)	Treatment plans can be digitally locked by selected users	Automatic connection between patient, prescription treatment plan and verification images
Automatic link of the treatment plan to the corresponding patient	Unlocked treatment plans can’t be applied to a patient	
Automatic link of the treatment plan to the corresponding target volume or clinical protocol respectively		
After radiation the treatment plan can’t be changed or deleted (only copies can be made)		
Positioning information is linked to the treatment plan		
Positioning information and patient photo can be displayed in the treatment room		
Remote control		
Periodical upgrades available, (with no data loss, for example user defined templates)		
High data integrity		
**Requirements for patient verification**		
**Minimal**	**Enhanced**	**Optimal**
Automatic calculation of the 3D correction vector of the treatment table (2D planar images and CBCT)	Automatic link of the verification images to the corresponding field of the treatment plan	Manual adjustment of the field edges of the verification field always possible
Automatic correction of the table by the system		
Automatic link of the verification images to the corresponding treatment plan and patient		
Correction data from offline analysis is automatically sent to the treatment system		

**Table 2:** Requirements for accuracy

**Data import, data registration and structure contouring**		
**Minimal**	**Enhanced**	**Optimal**
Import of external CT, MRI or PET data	Registration of any number of data sets	Flexible registration between CT and CT
Automatic rigid registration of 2 different data sets (e.g. CT and MRI) based on grey scale values	Automatic registration error measured in a phantom ≤ 0.5 mm	Flexible registration between different data sets
Registration of the planned CT with a combined PET-CT	Propagation of a structure to any registered data set	Flexible registration variable selectable in both directions
Automatic registration error measured in a phantom ≤ 1 mm	The propagated structure automatically receives a new name and / or a specific index	Automatic registration error measured in a phantom ≤ 0.2 mm
Structures can be copied to both sides between rigid registered data sets	Correction tools for example to cleanup pixels out of a selected VOI	Structures can be copied to both sides between flexible registered data sets
Structure from one dataset is representable in all registered data sets		
Boolian operations (AND, OR, NOT)		
Automatic expansion and contraction of structures with margins selectable in all three-dimensional directions		
Density override		
**Requirements for treatment planning**		
**Minimal**	**Enhanced**	**Optimal**
Conventional IMRT possible (step-and-shoot or sliding-window IMRT)	Volumetric IMRT possible (e.g. RapidArc or VMAT)	Volumetric IMRT with more than one arc and selectable segments
Create sum of treatment plans calculated on one data set	Direct manual manipulation of the fluences possible	Biological optimization and calculation
Use of more than one isocenter in one treatment plan	IMRT optimization with a DVH based declaration of the constrains	Flattening filter free mode planning
Non-coplanar fields are applicable (even for IMRT)	Dose and field entries and exits presentable on the body contour	Create sum of treatment plans calculated on flexible registered data sets
Display option of a structure in the BEV (e.g. for adjustment of saturation fields)	Reference dose can be applied to the treatment plan without linking to an anatomic location of the data set	Fit of isodoses to PTV or OAR by dragging the isodoses
Possibility of using a treatment plan as a base dose plan for a new optimization	TCP and NTCP model calculation included	
Convert an isodose to a structure		
Calculation and export of dose matrices (fluence) in transversal, sagittal and coronar slices		
Transfer of the fluence distribution on any CT data set and any phantom for the physical verification of dose		
Adjustable calculation grid		
**Requirements for patient verification**		
**Minimal**	**Enhanced**	**Optimal**
2D fluoroscopic images and CBCT images producible in the treatment room directly before any fraction		
Fast execution, high solution, good clinical image quality		
Image overlay between the DRR of the treatment plan and the verification images (2D planar images)		
Image overlay between the CT slices of the treatment plan and the verification images (CBCT)		
Automatic matching and manual matching possible		

**Table 3:** Requirements for efficiency

**Data import, data registration and structure contouring**		
**Minimal**	**Enhanced**	**Optimal**
2D brush, 2D pencil, 2D rubber	Possibility of choosing the CT slices, which should be imported (some treatment planning system are not able to handle too much slices), manually	Possibility of choosing the CT slices, which should be imported (faster treatment planning), manually
	Automatic contouring of the body contour	All structures are created automatically after selection of the treatment scheme
	Undo function	Propagated structures are automatically adapted to the new data set
		3D brush, deformable brush, structures stretchable, 3D rubber, structures can be drawn in sagittal and coronal slices
		Automatic segmentation of all organs at risk
**Requirements for treatment planning**		
**Minimal**	**Enhanced**	**Optimal**
IMRT optimization with a pencil beam algorithm	Optimization time for a common conventional IMRT <15 min.	Optimization time for a common conventional IMRT <5 min.
Automatic positioning of the leafs in a defined distance to the PTV	User defined DVH with an automatic display of OAR and PTV limits (green all well, yellow clinically acceptable, red out of limit, implementation of actual literature included and changes possible)	User defined print option using one button with possible inclusion of e.g. individual tables (Adaption of national laws)
Optimization time for a common Conventional IMRT <30 min.		Simple creation of QA plans or Service used plans (goal with one button)
		Clinical protocols for all tumor entities with automatically linked dose concepts, structure templates, OAR structure templates, OAR dose constraint templates, treatment plan and optimization templates
**Requirements for the ROKIS**		
**Minimal**	**Enhanced**	**Optimal**
License always available on every ROKIS workstation	Integrated software concept with treatment planning, treatment delivery, patient verification among others (except CT) in the ROKIS	QA Mode capability: Treatment of all treatment plans for QA purposes possible with corresponding rights
Specific and clearly understandable error messages	Fast system	
Anonymization of patient data included (e.g. for data export and clinical studies)	Possibility to open one or more sessions per workstation	
**Requirements for patient verification**		
**Minimal**	**Enhanced**	**Optimal**
Verification data correctable at any time after treatment (offline analysis possible)		

**Table 4:** Requirements adaptive treatment

**Requirements for accuracy**		
**Minimal**	**Enhanced**	**Optimal**
Automatic link of the CBCT images to the corresponding treatment plan and CT (the applied shift on treatment table must be integrated in the link)	Adaptive system automatically indicates if a new treatment plan is clinically needed (predefined limits by the user)	Adaptive system automatically creates a new treatment plan if clinically needed (predefined limits by the user)
**Requirements for efficiency**		
**Minimal**	**Enhanced**	**Optimal**
Automatic transfer from the CBCT images to the treatment system		
For online adaptive treatment: automatic propagation and adaption of the original structures and the original plan including sum plan and DVH of the sum		

**Table 5:** Requirements for respiratory gating

**Requirements for accuracy**		
**Minimal**	**Enhanced**	**Optimal**
Planning system supports 4D-CT data sets	IMRT technique used can be applied in gated mode	Automatic propagation of the structures between the different phases
Planning system supports the localizer used	Patient verification can be done with respiratory gating (CBCT)	Patient verification can be done with 4D-CBCT
Planning system supports amplitude gating and phase gating		Registration of 4D planning-CT with 4D-CBCT is possible
Register of diagnostic data sets to the 4D data set is possible		
Planning system can create an ITV out of GTV’s contoured in different phases		
Structure copy from one to another phase is possible		
Patient verification can be done with respiratory gating (2D planar radiographics)		
Data transfer of the chosen gating window possible between CT, planning system and treatment system		

For adaptive treatment most of the tools only have to met the minimal requirements gin in Table 4. These tools are available but still much too slow to be implemented in daily clinical routine. Respiratory gating is gaining in importance in the future (Table 5), if we can minimize or correlate the differences between interior and exterior patient movement. The systems available today from the radiation oncology vendors already meet most requirements.

## Discussion

The ability to assess the clinical applicability and compatibility of software tools is the most important requisite for smooth and fast processes in clinical work and is often underestimated. The technical features are described in detail by the vendor and an optimal clinical implementation is presupposed, but in many cases the integration into the clinical workflow is non-optimal. For the customer it is very difficult and time consuming to analyse all the tools in detail. Therefore we summarized the technical features required for a smooth clinical process, in order to help the customer to make the right software choice.

Automatic rigid registration of two different data sets based on grey scale values or with dicom coordinates should be implemented in the treatment planning systems. Of great importance is the ability of the software systems to registrate more than two data sets. One example is, for instance, the ability to register the planning CT of a patient with a head tumour to the T1-weighted MRI and in addition to the FLAIR (fluid attenuated inversion recovery) MRI. All treatment systems should be capable of dealing with combined PET-CT data, which not only compromise a high precision therapy treatment but also conventional treatments such as radiotherapy for patients with lung cancer. If the registration is not lockable this can result in serious treatment failures (Table 1). Meanwhile flexible registration, which is important for the accuracy (Table 2), is available, but often as an extra tool with higher costs. The automatic registration errors are not documented well by the manufacturers. At the time, only few evaluations were made concerning the technical uncertainties [2]. On that account we recommend an accuracy of the technical rigid registration uncertainties determined by phantom measurements of no more than 0.2 mm for an optimal treatment. Flexible registration in both directions (from CBCT to planning CT or vice versa) should ideally be implemented in the planning system in the future [3]. The significance seems to be high, but is not evaluated satisfactorily [2]. Generally the registration results and the registration time of the different systems should be analysed carefully.

A basic feature for the accuracy is the ability to copy a structure from one data set to another rigid registered data set (Table 2). This should be evaluated before purchasing a new system. Also the representation of one structure in another registered data set should be investigated carefully. Some software systems are able to create all necessary structures after selecting a tumor entity associated treatment scheme, which leads to a faster contouring process (Table 3). This is important for the efficiency of the system due to the automatic segmentation of the organs at risk [4]. This tool is available for most planning systems, but is very expensive due to the expanded research requirements. Optional contouring tools only accelerate the contouring process but do not lead to a better security [5]. The safety can be increased if the user can lock the structures manually (Table 1). Additionally the structures should be automatically locked digitally after connecting to a digitally locked treatment plan. It should be checked carefully, whether these tools are implemented in the system to ensure maximum safety.

The available planning features in modern treatment planning systems are mainly acceptable [6]. The attention should only be laid on the calculation algorithm only [7], which should either be a collapsed cone, Monte Carlo or an equivalent algorithm for best accuracy (Table 2). To create a sum out of any treatment plans (independent of the data sets) is the absolute basic tool and should be particularly emphasized. This tool should be queried before any decisions concerning the software are made, because, for example, a manual estimation of the total lung dose from two different treatment plans can result in large discrepancies between estimated and real applied dose. The software can solve these discrepancies. The possibility of using a treatment plan for a new optimization or to convert an isodose into a structure is a very useful tool for the assessment of the preload. This is important for the dose accuracy of retreated patients which becomes more and more important not only in the high precision therapy, but also in the normal clinical routine.

Currently more and more treatment plans with more than one target volume and only one isocenter or plans with an integrated boost are generated [8]. For these modalities new software features are needed. For treatment plans with an integrated boost, the possibility of connecting two different single and total doses to one treatment plan which both should be automatically added during the treatment series, is needed. For treatment plans with two anatomical separated target volumes (e.g. two parts of the spinal cord) it would be helpful to allow dose counting in both volumes separately. These features are important for patient safety (Table 1). But adding treatment plans can be dangerous if for example the fractionation is different and should be carefully analysed by the user. Further a possibility of presenting the field entries and exits on the body contour to check whether there is an overlap between the fields on the skin or not would be pleasant. This tool is also helpful for the analysis of the preload.

The ROKIS systems should be able to import treatment plans to archive them and to export them later on. But it is important to check if the exported treatment plan can be used again in the treatment planning system e.g. for sum plans with new treatment plans after export and reimport. Otherwise the archive can only be used for documentation. The best way is to have an integrated system of ROKIS, the treatment system and the data archive, which does not need to import and export any treatment plans. Such an integrated system is good for safety, accuracy as well as for efficiency. It also has the additional advantage that retrospective analysis of patient statistics, quality assurance and research can be done easily and comprehensively for the whole period of the system.

To have access to clinical templates with e.g. automatically linked dose concepts, structure templates, OAR structure templates, OAR dose constraint templates, treatment plan and optimization templates is very helpful for the clinical routine to improve the efficiency. Also treatment concept standardization can be integrated in the software to increase uniformity of treatment schedules.

It is essential that the treatment plan can automatically (!) be linked to the corresponding patient to avoid mistakes (Table 1). Even more important is the automatic link of the verification images to the patient, both the verification images of the treatment plans and the verification images from the treatment system. A link of the verification image to the corresponding field of the treatment plan would be even better. A permanent link between the patient, the clinical prescription, the treatment plan and the verification images is the most desired solution for optimal safety.

For patient verification nearly all functionalities must be declared as "minimal requirements". General patient verification is essential for high precision therapy [9]. It must be accurate, fast and as far as possible automatically efficient. Because of the high radiation exposure and the large time requirements for CBCT [10], the 2D planar imaging modality in the treatment room is also desirable [11]. The image modality must include direct image overlay for both modalities, because the error ratio of a layer overlay is too large. Whether automatic matching is more precise than manual matching is not demonstrated, but it is evidently faster. The demand of an automatic calculation of a 3D correction vector and automatic correction of the treatment table by the system is fundamental for the safety in high precision therapy.

For adaptive planning all "minimal requirements" for high precision therapy must be fulfilled (Table 4). Additional features such as automatic transfer from CBCT images to the treatment system with an automatic link of the CBCT images to the corresponding treatment plan and CT should be possible. Also important for offline adaption (Table 5) is the fact that registration between the CBCT and the planning CT automatically equates the actually applied shift. A prerequisite for this technique is the automatic propagation of the original structures and the original plan [12], including the calculation of an actual sum plan with DVH, which can also be helpful for offline adaptive planning [13]. Online adaption is still a vision for the future but not unachievable [14].

For all systems the inclusion of periodical upgrades is essential. The development of the software and the software options move rapidly and the upgraded systems are mostly faster and safer. But the most important point is the fact that software systems, which were not updated, may be not compatible to newly purchased software or hardware tools.

The tables should help the user to check the necessary and additional features, which should be integrated in modern treatment planning and ROKIS sytems. We recommend a point-based system with 10 points for all maintained minimal requirements, 5 points for all maintained enhanced requirements and 1 point for all maintained optimal requirements to receive a rating number for the software system.

## Conclusions

We analysed the features of different software systems, which are needed for a smooth clinical process in high precision radiotherapy treatment for a future customer. In particular the patient verification subsystem needs a detailed examination. All minimal requirements should be fulfilled. Other requirements will make the clinical processes faster or more precise.

## Competing interests

The authors declare that they have no competing interests.

## Authors’ contribution

HV, KZ and DMW carried out the technical analysis of the different treatment techniques. RE participated in draft of the manuscript. All authors read and approved the final manuscript.
